# EP31 Setting fire to sleeping giants: multiple autoimmune inflammatory diseases set off by chronic viral infection

**DOI:** 10.1093/rap/rkaa052.030

**Published:** 2020-11-03

**Authors:** Premila Kadamban, Jobie Evans

**Affiliations:** Addenbrooke's Hospital, Cambridge, United Kingdom

## Abstract

**Case report - Introduction:**

Autoimmune diseases are a group of disorders where the body produces an immune response against its own tissue constituents. The understanding of the immune mechanisms underlying autoimmunity has significantly deepened and broadened recently.

The viral infection is a well-known trigger of autoimmunity and chronic hepatitis is known to cause various auto-immune diseases; organ-specific or systemic.

Self-reactivity is part of the normal regeneration and healing. Auto-reactive T&B cells are kept dormant by the concerted action of a variety of mechanisms.

Hepatitis C can disrupt this by molecular mimicry, by providing adjuvant and even by directly affecting B cell function.

**Case report - Case description:**

We report a case of a 77 yr old Caucasian lady from South Africa who presented to Addenbrooke's hospital complaining of general decline, multiple constitutional symptoms, and low-grade fever for nearly 6 weeks. She did not have any infective symptoms. She had a past medical history of Type 1 DM, Hypothyroidism, Sjögren’s syndrome Osteoporosis and chronic hepatitis C infection which was treated 4 years ago. Family history was not significant, and she was a non-smoker.

She was found to be thin with a BMI of 18, pyrexical with a temperature 37-38'c; otherwise, examination findings were unremarkable.

Initial investigations revealed high inflammatory markers with an ESR of 99 and CRP of 120. WCC was within the normal range as were her renal and liver function tests. She was thoroughly investigated for Pyrexia of Unknown Origin. Her septic screen including Echocardiogram was negative. There was no detectable serum Para-protein and tumour markers were negative. Her CT scan of the chest abdomen and pelvis, MRI scan of the spine and PET scan were reported to be normal. The Serum Pro-calcitonin was low.

Rheumatology team was enlisted at this point. On further assessment, the patient mentioned of having an ongoing mild generalised headache - described as a 'fullness in the head' and mild generalised scalp tenderness. She also had an aching pain in her legs. There was no history of visual symptoms, jaw, or tongue pain. On examination, she had generalised scalp tenderness which was worse over the left temporal artery.

She had a temporal artery biopsy which showed typical features of GCA (disruption of IEL and giant cell) Following which she was commenced on steroids to which she had a remarkable response. After a month she was commenced on Tocilizumab with quick wean off steroids in view of her elevated risk for fractures.

**Case report - Discussion:**

This lady believes to have contracted Hepatitis C in the early 80s during a surgical procedure for removal retained products of conception following an incomplete miscarriage in South Africa.

She was found to be positive for hepatitis C in the early 90s when she tried to donate blood in the UK. She had genotype 1 hep C. on diagnosis her fibro scan was normal and live biopsy showed mild fibrosis. After regular surveillance during the following years, she was treated with AbbVie 3D regime and ribavirin in 2016 and she was declared to be clear in 2017 (Hepatitis C was undetectable).

This lady received a diagnosis of Sjögren’s syndrome in the early 2000s which was based on the fact that she had Sicca symptoms, Positive ANA of 9.2, positive anti-La and anti-Ro, Rheumatoid factor of 13 and lymphocytic clusters in lip biopsy. There are many studies linking Hepatitis C and Sjögren’s syndrome. Sicca symptoms are common in hepatitis C, as chronic sialadenitis is a well-known extrahepatic manifestation. A recent meta-analysis showed a strong association between Hepatitis C infection and Sjögren’s syndrome (OR3.31).

This lady was diagnosed with hypothyroidism in the early 90s. Thyroid autoantibodies were not checked then. Thyroid autoimmunity is quite common in Hepatitis C positive patients. Higher prevalence of anti-TPO (21%) and anti-thyroglobulin antibodies (17%) has been demonstrated in Hepatitis C positive population even before they develop hypothyroidism.

She was diagnosed with Type 1 Diabetes mellitus in 1997 at the age of 55 after she presented with Diabetic ketoacidosis. She was strongly positive for Anti-GAD antibody (>2000). Again, pancreatic autoimmunity is highly prevalent in non-diabetic Hepatitis C population (1.4% vs 0.4%). There are several case reports of patients who developed type 1 DM after acute hepatitis C infection.

**Case report - Key learning points:**

As chronic infections are known to trigger autoimmune diseases, it is advisable to screen for them in patients with multiple or atypical autoimmune diseases. Treating the infection itself will lead to complete remission of these autoimmune conditions.

